# Medullary Carcinoma of the Gastrointestinal Tract: Report on Two Cases with Immunohistochemical and Molecular Features

**DOI:** 10.3390/diagnostics11101775

**Published:** 2021-09-27

**Authors:** Cristina Colarossi, Marzia Mare, Giorgio La Greca, Marco De Zuanni, Lorenzo Colarossi, Eleonora Aiello, Eliana Piombino, Lorenzo Memeo

**Affiliations:** 1 Pathology Unit, Department of Experimental Oncology, Mediterranean Institute of Oncology, 95029 Viagrande, Italy; cristina.colarossi@grupposamed.com (C.C.); lorenzo.colarossi@grupposamed.com (L.C.); eleonora.aiello@grupposamed.com (E.A.); eliana.piombino@grupposamed.com (E.P.); 2 Medical Oncology Unit, Department of Experimental Oncology, Mediterranean Institute of Oncology, 95029 Viagrande, Italy; marzia.mare@grupposamed.com; 3 Department of Biomedical, Dental, Morphological and Functional Imaging Sciences, University of Messina, 98122 Messina, Italy; 4 Surgical Oncology Unit, Department of Experimental Oncology, Mediterranean Institute of Oncology, 95029 Viagrande, Italy; giorgio.lagreca@grupposamed.com (G.L.G.); marco.dezuanni@grupposamed.com (M.D.Z.)

**Keywords:** medullary carcinoma, colorectal carcinoma, small bowel carcinoma

## Abstract

Medullary carcinoma of the colon is a rare histological variant characterized by a poorly differentiated morphology, an aberrant immunophenotype, and microsatellite instability. Despite the lack of glandular differentiation, medullary carcinoma is reported to have a good prognosis. It is typically located in the right colon and frequently affects older women. Due to its clinical, histological, biological, and genetic peculiarity, medullary carcinoma requires an accurate diagnosis and the awareness of this diagnostic possibility. We describe the morphological, immunohistochemical, and molecular findings of two interesting cases, the first one in the right colon of a patient and the second one in the terminal ileum of a patient with Crohn’s disease. Deeper knowledge of all the biological and clinical features will allow appropriate and specific treatment of this tumor in the future.

## 1. Introduction

Colorectal cancer (CRC) is a heterogeneous disease. It is the third most frequently diagnosed cancer in men and the second in women, representing 10% of all tumor types worldwide [1]. More than 90% of CRCs are adenocarcinomas not otherwise specified (NOS) originating from epithelial cells of the colorectal mucosa [2]. Over 70% of CRC cases are sporadic, less than 10% of cases are inherited, and the remaining cases exhibit an increased familial risk [3]. Apart from the anatomical and morphological features, many genetic and epigenetic factors are responsible for the clinical diversity of colorectal carcinomas and represent important biomarkers for therapeutic decision. An international expert consortium has identified four molecular subtypes, defined as CMS1-4. The CSM1 group is characterized by microsatellite instability due to defective DNA mismatch repair and MLH1 epigenetic silencing and frequent BRAF mutations while having a low number of somatic copy number alterations (SCNAs). Moreover, the group shows strong immune activation. Patients with the CMS1 subtype have a poor survival rate after relapse. The remaining three subtypes, MSS stable, are linked to chromosomal instability (CIN) and show a high number of SCNAs and common mutations in the APC, TP53, KRAS, SMAD4, and PIK3CA genes [4]. Some of the identified mutations are necessary to enable targeted therapy. The MSI status helps clinicians in selecting adjuvant therapy. Moreover, the mutational status of KRAS, BRAF, and PIK3CA is taken into account for anti-EGFR therapy of metastatic CRC [5]. Since molecular events play a crucial role in prognosis and therapy decisions, and given the significant increase in CRC detection [6], it has become essential to identify and characterize the different subtypes of carcinoma. Medullary carcinoma is an uncommon histological variant morphologically characterized by a solid growth pattern with poorly differentiated or undifferentiated, non-glandular, solid sheets of eosinophil neoplastic cells with vesicular nuclei, prominent nucleoli, and abundant eosinophil cytoplasm usually associated with a significant amount of tumor-infiltrating lymphocytes [7]. The neoplastic cells show frequent loss of MLH1 and microsatellite instability associated with BRAF mutation (V600) [8]. Medullary carcinoma accounts for 2.2% of all colon carcinomas but represents about 20% of the poorly differentiated adenocarcinomas of the large bowel and can be sporadic or developed in patients with Lynch syndrome [9]. It is frequently located in the right colon, affects older female patients, and occurs with a more voluminous mass compared to adenocarcinoma NOS [10]. In addition, it appears to have a more favorable prognosis compared to high-grade colonic adenocarcinomas [11]. Rarely, it has been observed in the small intestine, with only five cases reported in the literature thus far [12]. In the past, medullary carcinomas were frequently reported as poorly differentiated adenocarcinomas [13]. Given the growing possibilities of targeted therapies, it is now necessary to define the different types of carcinomas, particularly those with particular inflammatory responses or molecular biomarkers. In this article, we report two different cases of medullary carcinoma, one in the large bowel of a patient and the other in the small bowel of a patient, and we describe the morphological and molecular features that pathologists and oncologists must be aware of in order to better stratify and treat these rare carcinomas.

## 2. Case Report 1

**Figure 1:**
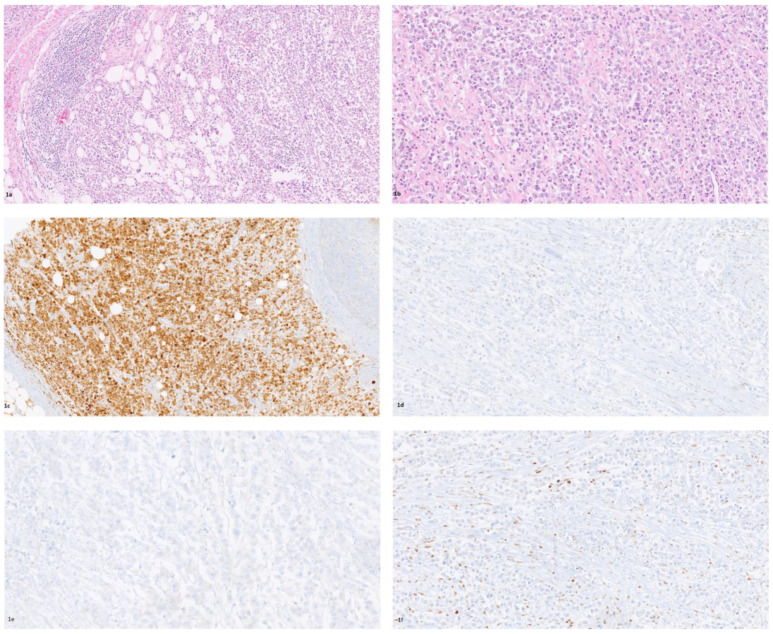
Neoplastic cells with abundant eosinophil cytoplasm and prominent nucleoli with intense intra- and peri-tumoral lymphocytic infiltration ((**a**) H&E 10×; (**b**) H&E 20×); diffuse and strong calretinin expression in tumor cells ((**c**) 10×); MLH1 loss ((**d**) 20×), CDX2 negativity ((**e**) 20×), and ARID1A loss ((**f**) 20×) in tumor cells.

A 70-year-old man presented with diffuse abdominal pain and the gradual onset of weakness in the preceding 2 months. There was no evidence of hematochezia or melena, with regular bowel movements; the patient referred to rare episodes of orthostatic vertigo that did not affect the quality of his life. No weight loss or loss of appetite was reported. The patient’s medical history included hypertension and diabetes mellitus; he used to smoke around 40 cigarettes per day since he was 20. He received an appendectomy when he was a teenager and thyroidectomy for multinodular goiter but had no family history of GI cancer. Once he was admitted to our hospital, a lab test revealed microcytic anemia, with Hb 7.2 g/dL and hematocrit of 26%. The CEA level was 2.25 ng/mL. A CT scan of the abdomen and pelvis was performed. The scan indicated colonic thickening of the caecum and the ascending colon, with adjacent fat stranding and caliber reduction of the colon. No lymphadenopathy or distant metastasis was reported (T3, N0, M0). Colonoscopy confirmed the presence of a lesion in the ascending colon and the caecum. Biopsies were taken but were not diagnostic. A laparoscopic right hemi-colectomy was performed based on the CT images of the colonic thickening of the caecum and the ascending colon and for the severe anemia. A voluminous mass was found in the caecum, with adhesions between the colon and abdominal wall and the last two ileal loops. The post-surgical course was uneventful, and the patient was discharged without complications 5 days after surgery. A gross examination of the surgical sample revealed a voluminous mass in the right colon, 8 cm in diameter, infiltrating the intestinal wall, with a trans-mural extension reaching the ileum. A histological evaluation showed a carcinoma with a solid architecture, and extensive sampling confirmed the lack of glandular differentiation. The cells displayed monotonous cytological features, with abundant eosinophil cytoplasm and prominent nucleoli. The intra- and peri-tumoral stroma was rich with lymphocytic infiltration (Figure 1a,b). Many necrotic foci were present. Immunohistochemical analysis showed a strong and diffuse expression of calretinin (Figure 1c), membranous beta-catenin, and negativity for CK20, CDX2 (Figure 1e), chromogranin, and synaptophysin. MMR analysis showed loss of MLH1 nuclear expression (Figure 1d) and PMS2. Moreover, loss of ARID1A was observed (Figure 1f). Molecular analysis performed with an automated Idylla platform showed microsatellite instability and BRAF mutation V600E in exon 15. Based on the morphological, immunohistochemical, and molecular features, colorectal medullary carcinoma was diagnosed. The patient started a chemotherapy regimen with oxaliplatin and capecitabine. Six months after surgery, the CT scan showed no recurrence of the disease, and the tumor markers were within the normal range.

## 3. Case Report 2

**Figure 2:**
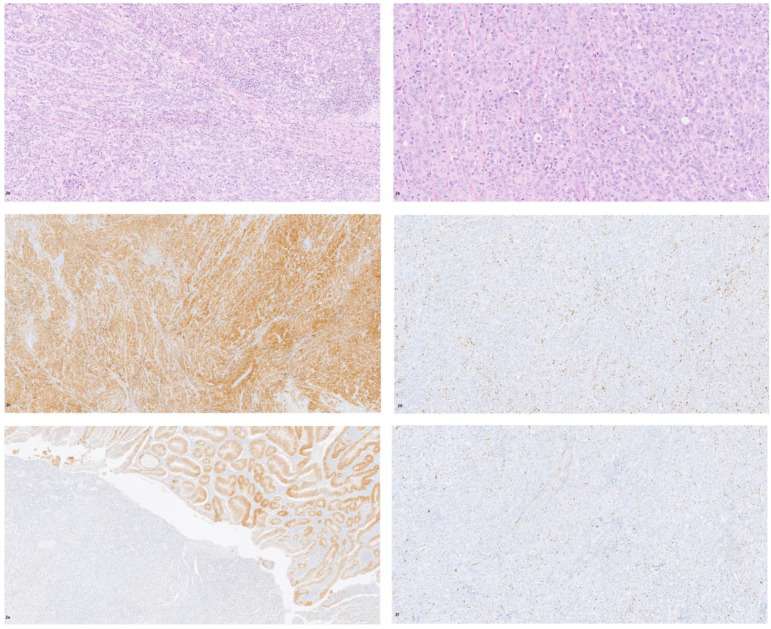
Solid and glandular architecture in mixed ileal adenocarcinoma–medullary carcinoma ((**a**), H&E 10×; (**b**) H&E 20×), with strong and diffuse calretinin expression in the medullary component ((**c**) 4×) and loss of MLH1 ((**d**) 10×) and CDX2 negativity in the medullary component, with diffuse expression in the glandular component ((**e**) 4×); ARID1A loss in the medullary component ((**f**) 10×).

A 62-year-old male patient, with a history of hypothyroidism, presented with a 3-week history of diffuse abdominal pain associated with nausea. Two months earlier, he had been hospitalized and discharged with the diagnosis of Crohn’s disease. On physical examination, severe pain was reported in the lower abdominal quadrants, with reduced peristalsis. Abdominal computed axial tomography showed small bowel obstruction with mural thickening and luminal narrowing of the terminal ileum. Routine laboratory tests showed WBC 13.2 × 10^3^ (neutrophils 75%), HB 13.1 g/dL, Hct 35.7%, and CRP 50. Tumor markers CEA, CA 19.9, CA 125, CA 15.3, and AFP were normal. The patient was transferred to the surgery division and underwent laparoscopic emergency ileocecal resection. The post-operative days were uneventful, and the patient was discharged on day 8 after surgery. Macroscopic analysis revealed a small bowel stenosis extending 6 cm. The mucosa showed several ulcerations and, in the stenotic tract, a white mass infiltrating up to the sub-serosal fat. Histological analysis of the surgical specimen revealed a tumor formed by two different histological components: a moderately differentiated adenocarcinoma NOS and a medullary carcinoma (Figure 2a,b). The medullary component shared all the histological and immunohistochemical findings of a colic tumor. The glandular component expressed CK20, CDX2 (Figure 2e), and ARID1A (Figure 2f) and was negative for calretinin (Figure 2c). The medullary component strongly expressed calretinin (Figure 2c) and was negative for CDX2 (Figure 2e), and CK20 and ARID1A (Figure 2f). Large areas of necrosis and inflammatory lymphocytic infiltrate were observed in the medullary portion. A mixed adenocarcinoma–medullary carcinoma was diagnosed. No regional lymph node metastasis was seen, and the tumor was classified as pT3, pN0, pM0 according to UICC. Immunostaining for DNA MMR proteins showed loss of MLH1 and PMS2, consistent with MSI-H (Figure 2d). Molecular analysis confirmed microsatellite instability and showed that KRAS, NRAS, and BRAF were wild type. Due to the poor clinical condition and low PS ECOG, no adjuvant chemotherapy was suggested. Six months after surgery, the CT scan showed no recurrence of the disease, the tumor markers were within normal ranges, and only routine follow-up was recommended.

## 4. Discussion

Medullary colon carcinoma, first described by Jessurun et al. in 1999 [14], is an uncommon tumor, representing a small percentage of all resected sporadic colorectal cancers [15]. The unusual morphology of this tumor type could be unrecognized, with the risk that its real incidence could be underestimated [16]. The lack of glandular differentiation can be misinterpreted as a poorly differentiated or undifferentiated adenocarcinoma NOS. Thus far, only a limited number of cases have been reported in the literature, and hopefully, more clinical and therapeutic information will be available in the future. Since the histological and molecular features are now well defined, pathologists must be aware of this diagnostic possibility. According to a recent edition of the WHO Classification of Gastrointestinal Tumors [17], medullary carcinomas display an aberrant phenotype, with loss of CDX2 and CK20; furthermore, these tumors show frequent microsatellite instability, with loss of expression of MLH1, and involve V600E BRAF mutation. Despite these defined characteristics, diagnosis can be challenging [18]. Additional studies have reported a more detailed characterization to improve the diagnostic algorithm. Winn et al. [19] explored an extensive panel including calretinin, CK7, CK20, p53, trefoil factor 3 (TFF3), MLH-1, MUC-1, and MUC2, in order to differentiate medullary carcinomas from poorly differentiated colonic carcinomas. In addition to the WHO guidelines, they observed a significant diversity of calretinin expression between the two categories, with 73% of the medullary carcinomas expressing the calcium-binding protein compared to only 12% of the poorly differentiated adenocarcinomas. Furthermore, MUC-1, MUC-2, and TFF3 expression was evaluated, showing that despite the loss of CDX2 expression, a significant portion of medullary carcinomas maintain intestinal differentiation. Further molecular studies proved that medullary carcinomas display loss of ARID1A expression [20]. Mutation of ARID1A is a recurrent event in colorectal carcinomas (CRCs), as reported recently by the Cancer Genome Atlas project (TCGA) group [21]. As previously observed for colon carcinomas deficient in MLH1, in medullary carcinomas, there is an association with ARID1A mutation and consequent loss of protein expression. In particular, this occurs in sporadic tumors harboring MLH1 hypermethylation [22].

Here, we report two cases of medullary carcinoma. The first one was a typical case, presenting as a large and infiltrating mass in the right colon of the patient, without lymph node metastasis. The eosinophil cells displayed solid growth without any glandular formation. The immunohistochemical pattern was coherent with the diagnosis and showed a strong expression of calretinin associated with a complete absence of CDX2 and CK20 expression. The mismatch repair status confirmed our diagnostic hypothesis, since MLH1 and PMS1 were lost, while MSH2 and MSH6 expression was intact. ARID1A was negative too, according to what was previously described. Molecular analysis showed V600E BRAF mutation and MSI. As previously reported, BRAFV600E mutation is significantly more common in medullary cancers compared with patients with non-medullary tumors and MSI [23]. The second case was a mixed form, with the glandular component presenting as a small bowel stenosis in the patient, who had Crohn’s disease. Medullary carcinoma is usually located in the large bowel while rarely being reported in the small bowel. Chronic inflammation is a predisposing condition in the development of small bowel carcinoma, and long-standing Crohn’s disease has been identified as a major risk factor. Small bowel tumors associated with Crohn’s disease are usually found in the ileum [24]. In the second case, in contrast to the colic neoplasia, the ileal tumor displayed some areas of glandular differentiation. Interestingly, only the medullary component showed expression of calretinin and loss of CDX2 and CK20, while the adenocarcinoma NOS component expressed CDX2 and CK20 and was negative for calretinin. Both components displayed loss of MLH1 and PMS2. Molecular analysis showed MSI-H and that BRAF was wild type. The case was signed out as mixed medullary adenocarcinoma, due to the dual morphological entities.

In both cases, we observed dense lymphocytic infiltration in the intra- and peri-tumoral stroma. Friedman et al. showed that the immune microenvironment of medullary carcinoma contains a higher mean of CD8+ tumor-infiltrating lymphocytes in epithelial and stromal compartments compared with microsatellite-unstable and microsatellite-stable tumors [25]. It has been shown that CD8+ tumor-infiltrating lymphocytes are stimulated by neo-antigens produced by microsatellite-unstable tumors and have been associated with a good prognosis [26]. All previous studies have confirmed that medullary carcinomas have a better prognosis compared to poorly differentiated and undifferentiated carcinomas. In particular, medullary carcinomas have a lower percentage of regional lymph node or distant metastases compared to G2 and G3 adenocarcinoma NOS. In addition, patients with medullary carcinoma showed a more favorable TNM stage distribution compared to patients with adenocarcinoma NOS [27]. A meta-analysis by Pyo [28] revealed that the overall survival rate of medullary carcinomas was significantly higher than that of poorly differentiated and undifferentiated adenocarcinoma NOS. Some of the prognostic biomarkers identified for adenocarcinomas have an unclear role in medullary carcinomas. In general, microsatellite instability (MSI) has been associated with favorable survival in early-stage colorectal cancers (CRCs) compared to microsatellite-stable (MSS) CRCs [29]. The V600E BRAF mutation confers a grave prognosis for stage II and stage III colon cancer patients [30]. Moreover, CDX2-negative colon carcinomas are associated with a lower rate of disease-free survival than CDX2-positive tumors [31]. The role of ARID1A mutation in colon cancer development and progression has not been fully clarified. The mutation is harbored in more than 50% of ovarian clear cell carcinomas, where it has been associated with grave prognosis in stage I and stage II carcinomas [32]. In colon carcinomas, the loss of expression of ARID1A has been associated with a bad TNM classification and high-grade morphology in some studies [33].

## 5. Conclusions

Medullary carcinoma has been recognized as an independent subtype of colorectal adenocarcinoma, from which it differs in clinical, histological, and prognostic aspects. To better understand the characteristics of this rare tumor and avoid mistakes in its grading and stratification, it is necessary to correctly identify and classify this subtype. We described the diagnostic parameters that allow the pathologist to distinguish medullary carcinomas from poorly differentiated/undifferentiated carcinomas. Moreover, we added the description of a rare case of small intestine medullary carcinoma to the literature. The limitation of this study is that it relied on a small number of cases, which does not allow a detailed molecular profiling of this neoplasm. Nevertheless, we think that it is important to draw attention to this under-recognized entity. Given the improvement in the pathological diagnostic criteria and the increasing clinical data available for oncologists, it may be presumed that patients with medullary carcinoma would receive more specific and accurate treatment in the near future.

## Data Availability

The data presented in this study are available from the corresponding author upon request.
